# Combinatorial approaches for mitigating resistance to KRAS-targeted therapies

**DOI:** 10.1042/BCJ20220440

**Published:** 2022-09-23

**Authors:** Hannah R. Warren, Sarah J. Ross, Paul D. Smith, Judy M. Coulson, Ian A. Prior

**Affiliations:** 1 Department of Molecular Physiology and Cell Signalling, Institute of Systems, Molecular and Integrative Biology, University of Liverpool, Liverpool L69 3BX, U.K.; 2 Oncology R&D, AstraZeneca, Cambridge CB2 0AA, U.K.

**Keywords:** drug resistance, G-proteins, signaling

## Abstract

Approximately 15% of all cancer patients harbor mutated KRAS. Direct inhibitors of KRAS have now been generated and are beginning to make progress through clinical trials. These include a suite of inhibitors targeting the KRAS^G12C^ mutation commonly found in lung cancer. We investigated emergent resistance to representative examples of different classes of Ras targeted therapies. They all exhibited rapid reactivation of Ras signaling within days of exposure and adaptive responses continued to change over long-term treatment schedules. Whilst the gene signatures were distinct for each inhibitor, they commonly involved up-regulation of upstream nodes promoting mutant and wild-type Ras activation. Experiments to reverse resistance unfortunately revealed frequent desensitization to members of a panel of anti-cancer therapeutics, suggesting that salvage approaches are unlikely to be feasible. Instead, we identified triple inhibitor combinations that resulted in more durable responses to KRAS inhibitors and that may benefit from further pre-clinical evaluation.

## Introduction

KRAS is one of the most frequently mutated oncogenes in cancer, and activating mutations are present in ∼2.5 million new cancer cases per year worldwide [1]. Mutant KRAS exhibits high prevalence in pancreatic, colon and lung cancers where it is typically an early lesion in the life history of the disease [2–4]. A spectrum of activating mutations are observed in cancer genetics databases [5], and it is increasingly apparent that the specific mutation present has prognostic and treatment implications [6]. The KRAS G12C mutation is associated with smoking-related mutagens [7], and is observed in ∼10% of non-small cell lung cancer cases [8].

Agents targeting the G12C mutation represent the first suite of direct KRAS inhibitors that show clinical efficacy. In pre-clinical models, they down-regulate KRAS- mediated MAP kinase activation and cause tumor regression in patient derived xenografts [9,10]. Several have progressed into clinical trials [11]; amongst these, Sotorasib (AMG-510) and Adagrasib (MRTX-849) are already showing high levels of disease control in early phase lung and colorectal cancer trials [12–14]. However, this is followed by acquired resistance to therapy and patients in these studies eventually show disease progression [12,14].

Resistance mechanisms to KRAS^G12C^ targeted therapies are beginning to be described in pre-clinical models and patient samples. These comprise alternative activating KRAS mutations, mutations that impede inhibitor binding and/or amplification or mutation of genes upstream or downstream of KRAS that result in re-activation of proliferative pathways [10,12,15–22]. Linked to these insights, combination therapies that can mitigate or delay emergent resistance are being trialed [23]. These primarily involve vertical Ras pathway inhibition via combining KRAS^G12C^ inhibitors with drugs targeting the ERBB family of receptor tyrosine kinases or SHP2 that promote Ras activation, or downstream effectors MEK and CDK4/6 that promote proliferation [23].

Anticipating that KRAS^G12C^ inhibitors would lead to drug resistance we generated a panel of drug resistant lung cancer cells where acute and chronic responses to treatment could be profiled. Unlike the studies described above, we wanted to compare and contrast resistance mechanisms amongst different classes of Ras pathway inhibitors. This included two KRAS^G12C^ inhibitors ARS1323 and ARS1620 that target the mutant protein but spare wild-type KRAS [24], the KRAS antisense oligonucleotide inhibitor AZD4785 [25] that will target both wild type and mutant KRAS, and the MEK1/MEK2 inhibitor selumetinib (AZD6244/ARRY-142866) [26,27] that targets a key downstream effector of KRAS. Our data reveal a diverse array of resistance signatures that converge to re-activate the Ras pathway and highlight how these therapies likely decrease the efficacy of other anti-cancer therapies targeting Ras network nodes. We also identify potential combination therapies that delay or mitigate emergent resistance.

## Results

### Generation of resistant cells

**Figure 1.**
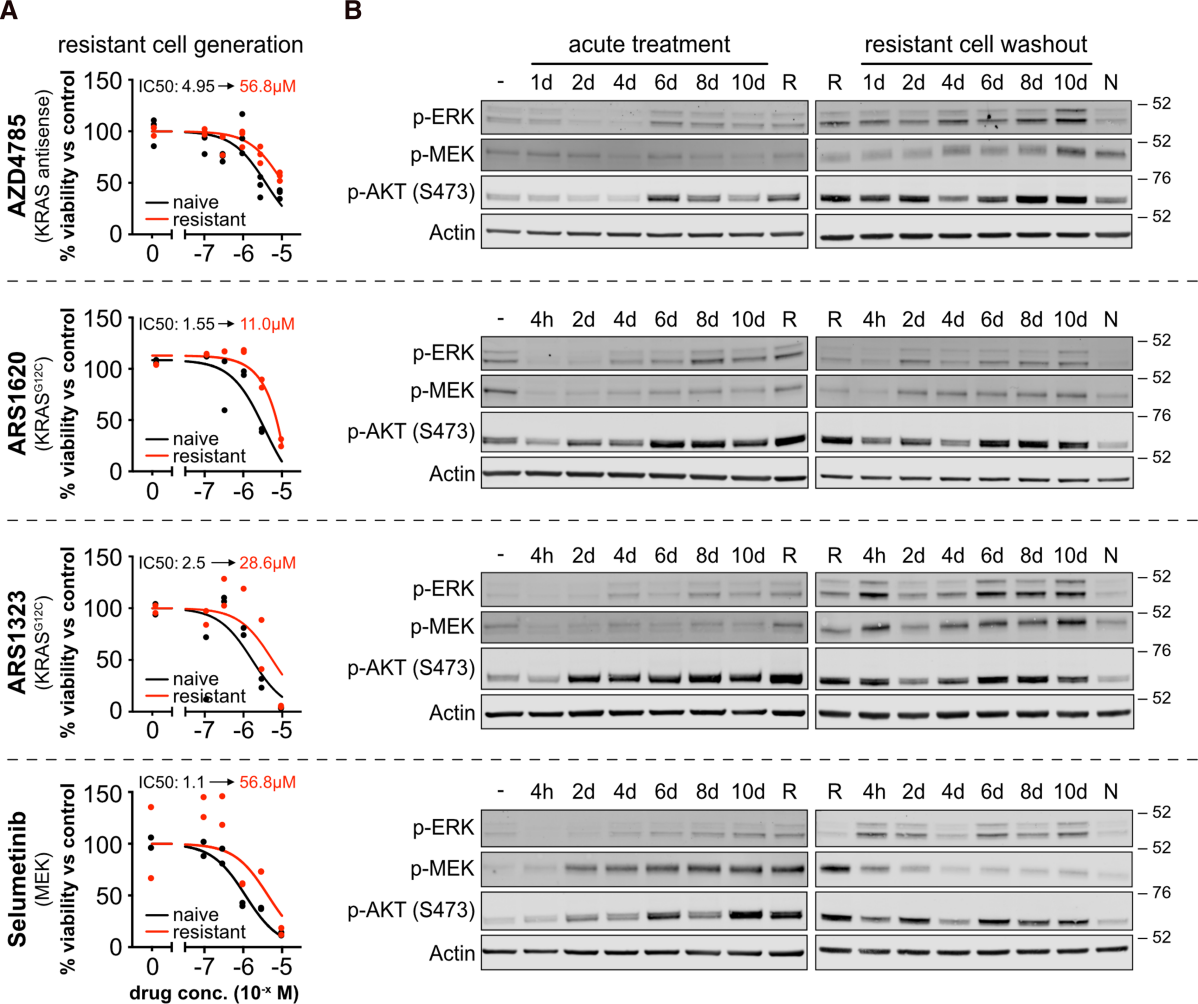
Resistance to Ras targeted therapies is rapidly induced and results in stable rewiring of Ras effector pathways. (**A**) Representative plots including technical replicate data points of drug responses in resistant vs naïve NCI-H358 cells. (**B**) Acute treatments of naïve cells with IC_90_ concentrations of inhibitor re-dosed every 48 h results in rapid re-activation of Ras effectors. Where re-dosing or cell harvesting occurred on the same day, e.g. 4 days, cells harvested at day 4 will have been re-dosed 48 h previously. High levels of Ras pathway activation are maintained in resistant cells even after drug washout with vehicle. (−) cells treated with vehicle control, (R) resistant NCI-H358 cells continuously cultured for at least 30 days in the presence of drug, (N) naïve NCI-H358 cells. Blots are representative of *n* = 3 biological repeats.

The NCI-H358 non-small cell lung cell line harbors a heterozygous KRAS G12C mutation. NCI-H358 cells exhibit sensitivity to our panel of Ras pathway inhibitors, with both 3D viability and pathway inhibition in 2D culture typically occurring at ∼10-fold lower concentrations than seen for effects on viability in 2D culture (Supplementary Figure S1). Long-term culture with IC_90_ inhibitor concentrations, was used to generate resistant NCI-H358 cells with at least a 5-fold reduction in sensitivity to drug (Figure 1A). In each case, resistance is associated with reactivation of the Ras-MAP kinase pathway to levels equivalent to vehicle control, whilst Ras-PI3 kinase signaling exhibits a significant increase over control (Figure 1B). These cells have experienced up to 100 days in the presence of inhibitor with consistent responses. However, acute treatments revealed that these resistance-associated signatures are evident within a few days of exposure to drug (Figure 1B). Drug washouts in chronically resistant cells did not restore Ras signaling to levels seen in naïve cells suggesting permanent rewiring associated with adaptive resistance (Figure 1B). Rapid reactivation of Ras outputs and permanent rewiring of resistant cells were common to all classes of drug that we tested. It is notable that whilst MAPK pathway nodes are variably up-regulated across the panel, in all contexts the PtdIns 3-kinase (PI3K)-AKT response is consistently much higher than that seen in naïve cells, and permanently induced. Patterns of rapid MAPK and PI3K pathway reactivation were similarly observed in NCI-H1792 non-small cell lung cancer cells also harboring a heterozygous G12C mutation (Supplementarty Figure S2). In contrast, this was not observed in wild-type KRAS NCI-H1793 cells treated with direct Ras inhibitors. They all exhibited short term enhancement of AKT phosphorylation that may indicate a stress response; however, sustained rebound activation of MEK activation was only observed following selumetinib treatment. This suggests that these wild-type cells are sensitive to MEK inhibition and recovery but largely insensitive to the direct Ras inhibitors.

### Comparison of differentially expressed genes

**Figure 2.**
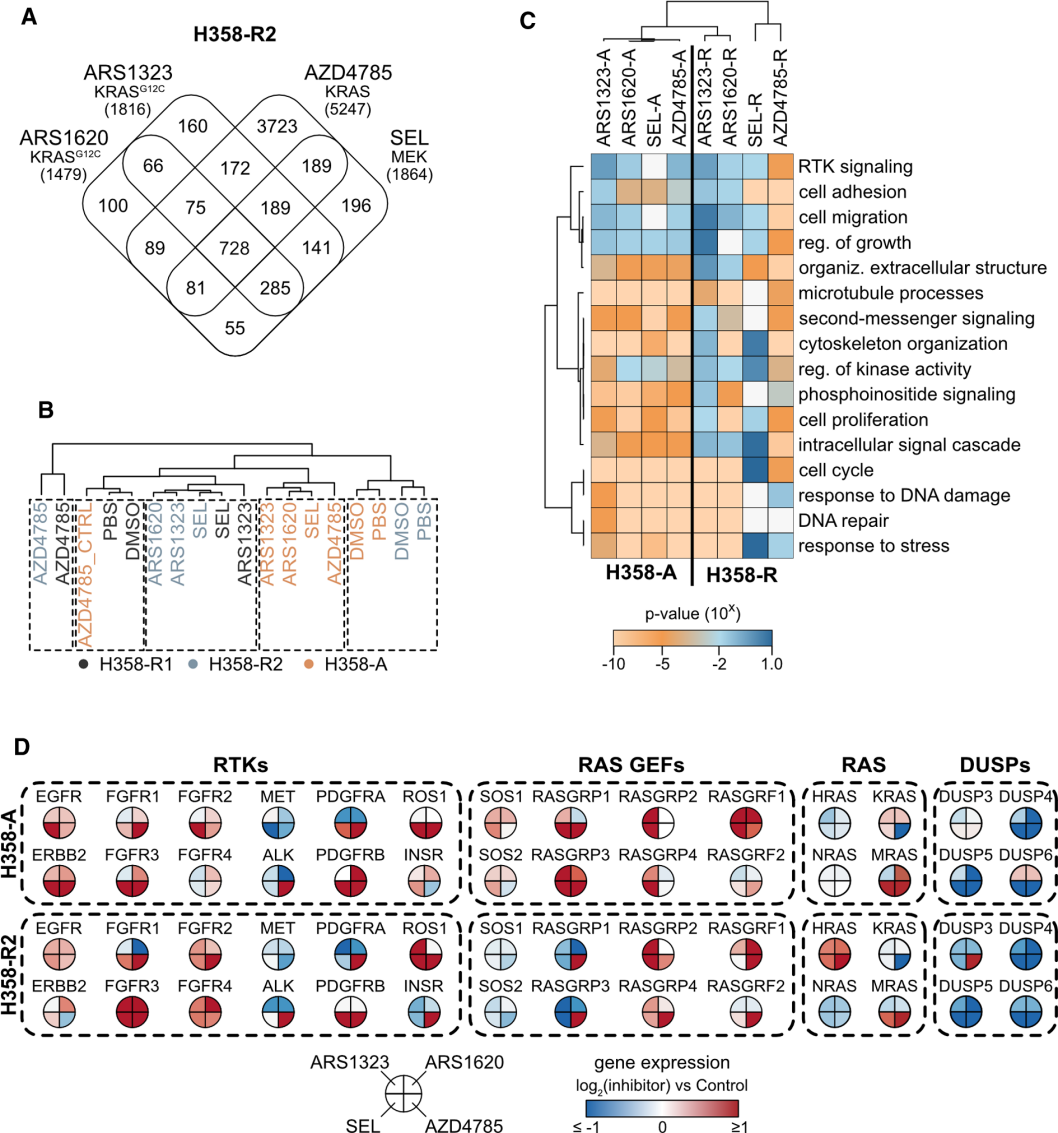
Gene expression responses associated with cells resistant to acute versus chronic exposure to KRAS/MEK inhibitors. Two sets of NCI-H358-R samples were processed independently (NCI-H358-R1 and NCI-H358-R2). NCI-H358-R2 samples were processed alongside NCI-H358-A samples. DMSO was the control comparator for ARS1620, ARS1323 and SEL, PBS was the control for AZD4785 in long term resistant cells whilst induction of acute resistance to AZD4785 used a biologically inert scrambled oligo (AZD4785_CTRL) as a control. Long-term resistant cells were processed for RNAseq analysis 90 days post initiation of resistance. (**A**) Overlapping but distinct differentially expressed gene (DEG) responses are observed in cells resistant to different classes of Ras pathway inhibitors DEGs are identified using a Poisson distribution algorithm where the following parameters are satisfied: ≥2-fold change versus control, false discovery rate of ≤0.001. The total number of DEGs is shown in brackets for each condition. Hierarchical clustering based on DEG responses (**B**) and GO terms (**C**) indicates significant differences between NCI-H358-A and NCI-H358-R cells with a tendency to normalize responses to the small molecule inhibitors over long-term treatment. All biological repeats used for transcriptomics analysis are depicted in the dendrogram (**B**). (**D**) Nodes that activate the Ras pathway are up-regulated in cells resistant to KRAS and MEK inhibitors.

To explore potential resistance mechanisms to KRAS inhibition we utilized RNAseq transcriptomics to identify differentially expressed genes (DEGs) in drug treated NCI-H358 cells versus their respective vehicle-treated controls. We profiled two independently derived populations of NCI-H358 cell lines resistant to chronic treatment (NCI-H358-R1 & NCI-H358-R2) and a set of acutely treated cells (NCI-H358-A). NCI-H358-A cells were exposed to the indicated inhibitor for 7 days, a timepoint when emergent resistance is clearly seen in all measured Ras outputs (Figure 1B). The AZD4785 antisense KRAS inhibitor generated a far larger number of uniquely expressed DEGs compared with the small molecule compounds (Figure 2A). There was a large overlap in DEGs across the cell lines resistant to KRAS and MEK small molecule inhibitors. Almost half of the DEGs observed in cells resistant to each of the small molecule inhibitors were common to all four inhibitors and 70% of the DEGs observed with the cells resistant to KRAS^G12C^ small molecule inhibitors were also observed in selumetinib resistant cells. Hierarchical clustering of DEGs from all three data sets demonstrated that resistant cells are clearly different from vehicle-treated controls (Figure 2B). Notably, the biological repeats (R1 and R2) co-clustered with each other indicating largely similar gene expression responses. There was a clear difference in the responses of chronically resistant cell populations (R1 and R2) versus acute treatments.

Responses to short term inhibitor exposure seem to be largely equivalent across the inhibitor panel. The differences between the inhibitors were most clearly seen following long-term treatment, with AZD4785 antisense KRAS inhibitor diverging from the other treatments (Figure 2B). Gene Ontology (GO) analysis of DEGs in each resistant cell population identified many enriched pathways associated with Ras-MAPK biology (Figure 2C). Focusing specifically on the Ras signaling network, we consistently observed increased expression of many upstream activators of Ras in both acute and chronically resistant groups (Figure 2D). This would favor increased GTP loading of KRAS^G12C^ leading to reduced G12Ci binding and would also promote KRAS-independent activation of Ras networks via the other Ras isoforms. Significantly increased HRAS expression is also observed in all chronically resistant cell populations consistent with the idea of resistance-associated Ras switching. Increased MRAS expression was also observed, MRAS is a member of the RAS superfamily that forms a complex with SHOC2 and PPP1CA to promote activating dephosphorylation of RAF1 [28]. This complex is known to mediate resistance to MEK inhibition [29]. We also observed downstream reactivation mechanisms associated with both acute and chronic resistance. DUSP family members that are responsible for negative feedback control of the RAF-MAP kinase network [30] were consistently down-regulated in response to Ras and MEK inhibition.

**Figure 3.**
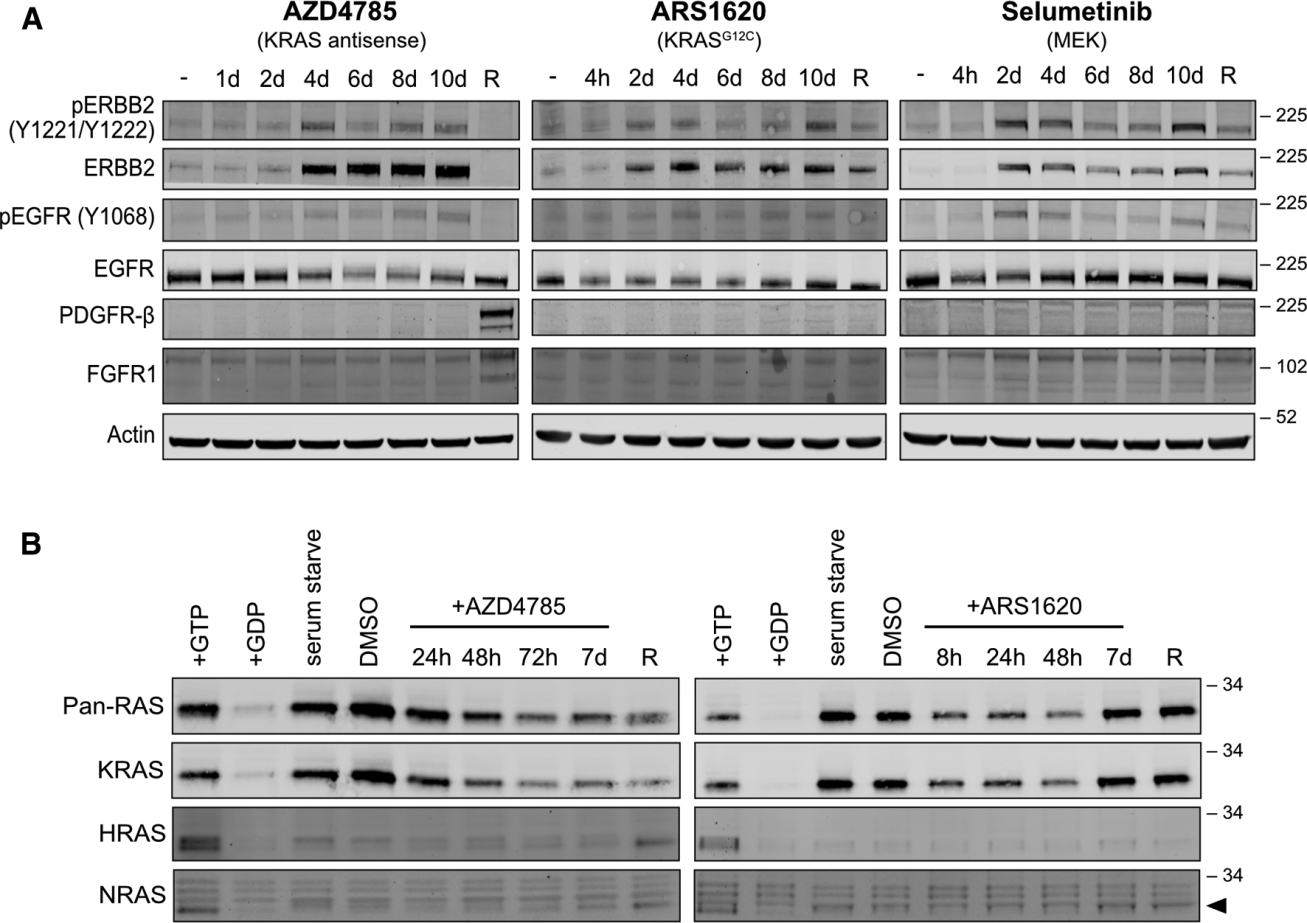
RTK expression and Ras activity can be rapidly remodeled in response to inhibitor treatment. (**A**) Dynamic and inhibitor-specific changes in RTK expression are observed over an acute inhibitor time course and in long-term resistant NCI-H358 cells (R). (**B**) KRAS activity is rapidly restored when cells are dosed with KRAS^G12C^ or MEK inhibitors. Long-term exposure to MEK inhibitor (AZD4785, R) also results in up-regulation of HRAS activation. In all cases naïve NCI-H358 cells were treated with vehicle control (−) or IC_90_ concentrations of each inhibitor for the indicated time periods with cells re-dosed every 48 h. Blots are representative of *n* = 3 biological repeats.

Both ARS1323 and ARS1620 KRAS G12Ci generated largely similar responses (Figure 2). They are both part of a related compound family [24], and since ARS1620 represents the more refined version [11], we used this in our subsequent analysis. The increased expression of EGFR, FGFR and PDGFR family members variously seen in both acute and chronically resistant cells (Figure 2D) were especially evident in response to AZD4785. These patterns of responses were confirmed at the protein level (Figure 3). Increases in ERBB2 protein occur within the first few days of exposure to inhibitor and stabilize at this high level (ARS1620) or partially (selumetinib) or fully (AZD4785) return to normal in chronically resistant cells. Higher levels of ERBB2 correlate with increased detection of activated ERBB2 with a phospho-antibody. Similar patterns to this are observed for activated EGFR but not for total EGFR protein. Significantly increased PDGFR and FGFR protein levels were only observed in cells chronically resistant to AZD4785. Therefore, whilst increased receptor tyrosine kinase expression and activation are a consistent feature of all inhibitor responses, the timings and preferred proteins are unique to each inhibitor.

We investigated whether KRAS inhibitor resistance is associated with reactivation of KRAS and/or bypass activation via other Ras isoforms (Figure 3B). For ARS1620 G12Ci, the initial declines in KRAS activation are rapidly reversed by 7 days of treatment to levels seen in untreated cells and long-term resistant cells. For the selective antisense KRAS inhibitor AZD4785, KRAS activation remains low. This is consistent with continued effects on maintaining lower levels of KRAS protein expression. In cells resistant to long-term treatment with AZD4785, higher levels of HRAS gene expression are observed (Figure 2), and this correlates with increased levels of HRAS activation (Figure 3B). In contrast, there is no evidence of bypass Ras activation in ARS1620 resistant cells since KRAS is already efficiently reactivated.

### Therapeutic combinatorial strategies

**Figure 4.**
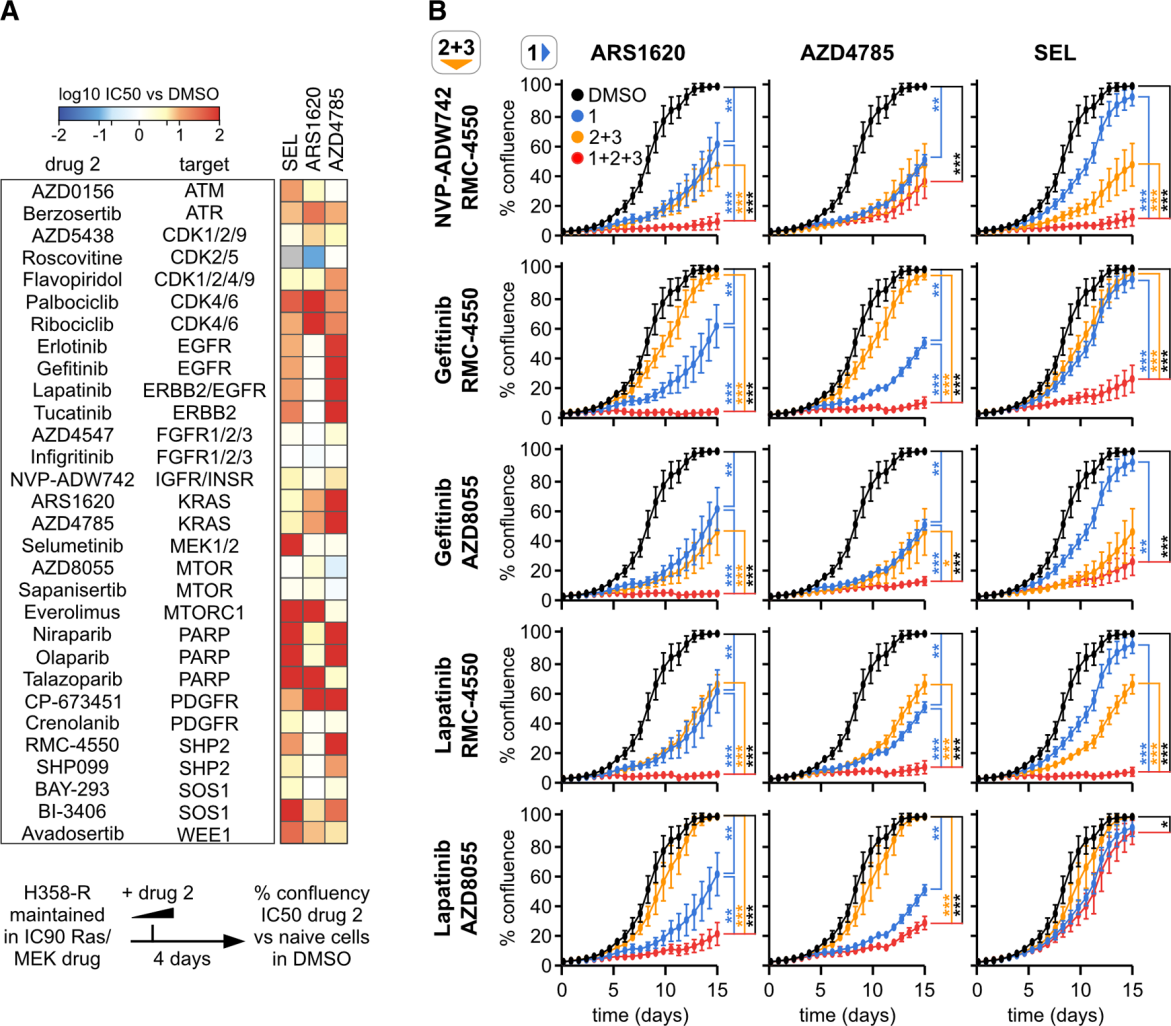
Combination treatments to mitigate Ras resistance. (**A**) Dose responses of combination therapeutics in resistant cells. Resistance associated with long term Ras pathway inhibitor dosing often results in desensitization to members of a panel of anti-cancer therapeutics versus DMSO control cells not exposed to Ras/MEK inhibitor. Dose responses are shown in Supplementary Figure S3. (**B**) Effective triple inhibitor combinations to mitigate emergent resistance to targeted therapeutics. RTK-KRAS-SHP2/MTOR inhibition delays resistance to KRAS therapeutics. Naïve NCI-H358 cells were treated with the indicated inhibitor combinations targeting KRAS (ARS1620, AZD4785), MEK (selumetinib), EGFR (gefitinib), EGFR/ERBB2 (lapatinib), IGFR/INSR (NVP-ADW742), SHP2 (RMC-4550) and mTOR (AZD8055), re-treated every 5 days and cell growth monitored using an IncuCyte S3. Data are from triplicate repeats of four independent experiments; statistical significance was evaluated using a Mann–Whitney test where * *P* < 0.05; (**) *P* < 0.01; (***) *P* < 0.001.

Identification of suitable inhibitor combinations will be crucial to maximizing the therapeutic potential of targeted KRAS inhibitors. We collated 25 drugs and inhibitors with known anti-cancer clinical efficacy and that target nodes that were consistently altered or associated with enriched GO terms in our resistant cells. We performed a preliminary screen to test them for their ability to overcome KRAS inhibitor resistance in our long-term treated cells (Figure 4A, and Supplementary Figure S3). In all but two cases, cells resistant to KRAS inhibitors responded the same or worse to the second targeted therapy. An example of cross-resistance is seen in cells resistant to AZD4785 that are more resistant to ARS1620 than ARS1620-resistant cells (Supplementary Figure S3). However, this does not seem to extend to the down-stream effector MEK where both ARS1620 and AZD4785 resistant cells remain sensitive to selumetinib (Figure 4A and Supplementarty Figure S3). Across the panel of combinations, rewiring associated with KRAS inhibitor resistance resulted in >10-fold increase in IC_50_ in 40% of the combinations. Therefore, pre-exposure to long-term Ras pathway inhibition frequently results in reduced efficacy of subsequent dosing of a wide range of other anti-cancer targeted therapies.

Preventing or delaying the onset of resistance is preferable to salvage treatment of resistant cells. To try to achieve this we used a triple combination strategy to improve vertical inhibition within the KRAS signaling network (Figure 4B). A triple combination strategy was selected over a double combination because it reduces selective pressure on each targeted node and is therefore more likely to delay the onset of resistance [31]. It also means that lower effective doses may be achievable for each inhibitor, reducing associated toxicity. KRAS/MEK inhibitors together with EGFR, ERBB2, IGFR, MTOR and SHP2 inhibitors were tested in naïve NCI-H358 cells for their effects on retarding cell proliferation. Doses for these combinations were selected following Loewe synergy analysis (Supplementary Figure S4). Incubation with either of the KRAS inhibitors resulted in a significant delay to population growth compared with DMSO control treatments (Figure 4B). Cells rapidly adapted to selumetinib treatment such that cell confluence was equivalent to control by the end of the experiment. Triple combinations delayed resistance to KRAS and MEK inhibitors in almost all cases. The KRAS inhibitors are more effective in combination than selumetinib. Three of the five ARS1620 triple inhibitor combinations exhibited complete inhibition of cell growth over the 15 days of the experiment with one of the pairs (Lapatinib + RMC-4550) strongly suppressing cell growth across all three KRAS/MEK inhibitor combinations. In summary, most vertical pathway inhibition triple combinations prolong the effective inhibitory time window compared with single KRAS/MEK inhibitor treatment, with RTK-KRAS/MEK-SHP2 inhibition proving to be consistently good in almost all contexts.

## Discussion

Excitement around the clinical efficacy of KRAS^G12C^ inhibitors is inevitably tempered by the observation of emergent resistance that is a common feature of drugs targeting this pathway. We observed rapid reactivation of Ras signaling in response to a panel of Ras targeted therapies with different modes of action. This was evident within only a few days of treatment and was consistent with previous observations of rapid adaptive responses to the KRAS^G12C^ inhibitor ARS1620 and the MEK inhibitor selumetinib [18,32]. The common patterns of reactivation seen with inhibitors that variously targeted only the mutant Ras protein, that targeted both mutant and wild-type KRAS or that targeted a key Ras effector showed the strong selective pressure for cancer cells to re-establish signal flow through the Ras network. The fact that these inhibitors are clinically effective despite such quick restoration of apparent Ras activity suggests more nuance is needed in the assessment of resistance.

Our study focussed on two independently derived H358 drug-resistant cell populations. Hierarchical clustering indicated that the similarity between the biological replicates was greater than the similarity between different inhibitors suggesting a degree of specificity in the response to each inhibitor. The stochasticity in response apparent between replicates together with the variety of responses observed in each cell population indicate that there isn't a stereotypical response that is consistently induced and speaks to subtle cell state and epigenetic differences that will select for mechanisms from a range of related options. This is analogous to the lack of a single resistance mechanism to a targeted therapy in patients where cell type and heterogeneity in cell state determine the responses that become selected during cancer evolution [33,34]. The underpinning influence of cell clonality in leading to a cancer cell population exhibiting a range of resistance responses was clearly shown recently in a group patients that became resistant to sotorasib (G12Ci) [35].

Across our panel of resistant cell populations we observed comprehensive up-regulation of RTKs and Ras GEFs and down-regulation of DUSPs. The effect of these changes will be to promote the activation of Ras isoforms and the Ras network that we saw. Most of these genes have been variously shown to mediate adaptive resistance to KRAS^G12C^ and MEK inhibitors [12,17–20,36,37]. In our comparison of different Ras targeted inhibitors, the reactivation mechanisms observed were qualitatively the same across all classes. However, there was significant inhibitor-specific heterogeneity in the constellation of resistance-associated genes. This, together with previously observed heterogeneity of resistance responses between cell types and within cell populations [18,36], means that there aren't generic solutions to preventing or overcoming emergent resistance.

We compared resistance associated with acute versus long-term treatment and observed a dynamically changing landscape of responsive genes that underscore the difficulty in identifying mitigating strategies even for a single inhibitor context. This included targets of drugs currently used to treat cancers where KRAS is frequently mutated. For example, ERBB family members are targets of gefitinib and erlotinib that have been used in first-line lung cancer treatment [38]. ERBB family members exhibited rapid protein or activation-reporting phosphosite up-regulation in response to KRAS inhibitors suggesting that they may be sensitive to co-inhibition of these upstream nodes as we explored later in our triple inhibitor combinations. However, these changes were most pronounced response to acute treatment and were subsiding in chronically KRAS inhibited cells suggesting a limited window of opportunity for co-inhibition.

Resistance to targeting a particular Ras variant could be mediated by bypass activation of either the wild-type allele in the case of the G12Ci and/or the other Ras isoforms HRAS and NRAS. Interestingly, this was only observed in some contexts. Specifically, we observed a switch to HRAS with all inhibitors only following chronic treatment. This was observed at the transcript level for all treatments and increased HRAS activation was observed in cells exhibiting long-term resistance to AZD4785 where both wild type and mutant KRAS are targeted. It is intriguing that NRAS was not similarly responsive given that it can also activate the same canonical pathways as KRAS and HRAS. The reason for this difference may be that HRAS is normally expressed at low levels [39]; and therefore represents the Ras isoform most capable of responding with large increases in expression. It is also druggable, farnesyl transferase inhibitors (FTIs) that mislocalize HRAS are showing promising results in clinical trials on HRAS mutant cancers [40]. Coincidentally, adaptive resistance to the FTI tipifarnib also results in Ras switching via loss of the Ras GAP NF1, resulting in enhanced activation of KRAS [41]. Therefore, combination therapies specifically targeting KRAS and HRAS may represent an optimal strategy for mitigating bypass resistance to both sets of Ras-targeted therapies.

Despite the challenges associated with resistance heterogeneity, targeting upstream reactivating mechanisms represents an attractive strategy. Encouraging results have been observed using combination approaches of RTK, SOS1 or SHP2 inhibitors to sensitize or prolong responses to KRAS inhibitors in naïve cells [9,10,15–17,42–44]. We explored whether this strategy could be applied to salvage treatment after resistance has already emerged. The type of KRAS inhibitor determined whether this was likely to be successful. Antisense KRAS (AZD4785) behaved like selumetinib in resistant cells showing desensitization to RTK and SHP2 inhibitors. Whereas in almost every case ARS1620 resistance did not negatively impact subsequent responses to combination therapies that include RTK or SHP2 targeting drugs. This may reflect the mechanism of action of ARS1620 versus the other inhibitors. ARS1620 binds to GDP-bound KRAS^G12C^ and spares wild-type KRAS [24,45]. Resistance associated with mechanisms promoting GTP-loading of KRAS^G12C^ or bypass activation of wild-type Ras are therefore likely to remain sensitive to inhibition.

Cross-resistance to Ras therapies was also observed. Cells resistant to AZD4785 were even more resistant to ARS1620 than that ARS1620-resistant cells were. Cells resistant to either of these inhibitors were still sensitive to selumetinib suggesting that salvage treatment may be possible with MEKi following the emergence of resistance to direct KRAS targeted therapies. Further work is needed to explore this. Whilst different classes of direct Ras inhibitor cannot be used to overcome resistance, there may be benefit in employing combinations of these classes to delay the onset of resistance. Although AZD4785 did not progress through clinical trials, there are a variety of inhibitors under development designed to induce Ras protein degradation or to bind to mutant Ras and inhibit effector interactions.

Although salvage treatment may be possible in some cases, in 40% of combinations we observed at least 10-fold desensitization to the second inhibitor. Therefore, combination therapies and treatment scheduling that increase the durability of the initial response to KRAS inhibitors are preferable. Targeting multiple components within a biological circuit at once can significantly improve outcomes [46], and vertical pathway inhibition was effective in mitigating KRAS inhibitor resistance [17]. More complex strategies involving triple combinations with everolimus (mTORi) and linsitinib (IGF1Ri) increased the efficacy of ARS1620 resulting in profound loss of KRAS^G12C^ mutant cell viability and regression of xenograft tumors [43]. We recapitulated this combination using alternative inhibitors and saw similarly effective inhibition with ARS1620. Intriguingly however, this combination did not show extra efficacy when the antisense KRAS inhibitor was added and did not delay resistance onset. We explored other triple strategies and identified SHP2i or mTORi plus EGFR/ERBB2i as highly effective resistance delaying combinations when used with either of the KRAS inhibitors. Although we didn't test SOS1 inhibitors, we speculate that they would phenocopy the SHP-2 inhibitor and further work to explore this in a salvage combination would be desirable. Finally, our combination studies focussed on inhibitors of upstream regulators promoting resistance-associated Ras re-activation. Triple combinations that include downstream effector inhibitors may also be efficacious. We particularly focussed on the RTK-MAPK pathway in the design of the triple combinations; however, we also saw a consistent up-regulation of AKT phosphorylation in all contexts. Recent work directly compared AMG510 (G12Ci) combinations with either MAPK or PI3K inhibitors and found that the MAPK combination was much more effective [9]. More generally, there may be some benefit in a triple combination format where lower individual doses might mitigate some of the toxicity seen with combination therapies targeting MAPK and PI3K pathways at the same time [47,48]. Although we did not investigate it in our work, there may also be benefits from exploring intermittent dose scheduling of members of double or triple combinations to forestall the onset of resistance [46,49,50].

In summary, we observed rapid onset resistance to all KRAS inhibitors that resulted in reactivation of the Ras pathway as well as upstream nodes. Taken together, our data highlighted the heterogeneity of resistance responses across inhibitors targeting the same pathway to achieve qualitatively similar results. Following the rapid emergence of resistance we also observed how it then remodels over time, often resulting in a state that will be desensitized to other cancer targeting therapies. To avoid these undesirable consequences we found that combination strategies will be more effective if deployed upfront rather than as salvage treatment. Triple combination approaches to mitigate adaptive resistance to KRAS inhibitors are described that are promising strategies for further evaluation in pre-clinical models for tolerability and efficacy.

## Materials and methods

### Cell lines

Authenticated NCI-H358, NCI-H1792, and NCI-H1793 cell lines were purchased from ATCC. NCI-H1793 harbor homozygous wild-type KRAS, both NCI-H358 and NCI-H1792 are heterozygous for KRAS^G12C^; KRAS is not amplified in any of the cell lines [51]. Cells were grown in RPMI 1640 Medium supplemented with 10% fetal bovine serum (FBS). Cells were cultured at 37°C in a humidified incubator at 95% O_2_ and 5% CO_2_ and were routinely passaged at 80% confluency. All cell lines were confirmed negative for mycoplasma.

### Cell culture compound treatments

Small molecule inhibitors supplied by AstraZeneca or purchased from Selleck were dissolved in dimethyl sulfoxide (DMSO) to yield 30 mM or 10 mM stocks and stored at −80°C. Inhibitors were stable at −80°C for 6 months or −20°C for 1 month. The antisense oligonucleotides AZD4785 and AZD549148 (AZD4785_CTRL, [25]) supplied by AstraZeneca were dissolved in phosphate-buffered saline (PBS) to yield 10 mM stocks and stored at 4°C with stability under these conditions beyond 1 year. Drug stocks were diluted in growth medium to provide a dosing solution that was applied to cells to yield the final drug concentration. Drug dilutions were prepared to ensure each well contained a final vehicle concentration of 0.1%. Growth medium containing vehicle-only (DMSO or PBS) was used as a control. Cells were treated in triplicates or as otherwise stated and incubated at 37°C at 95% O_2_ and 5% CO_2_ for the length of time indicated in experiments. To identify optimal combination doses, synergy scores from double inhibitor combination assays were calculated using the Loewe model and plotted against dose responses (% viability).

### Generation of resistant cells and drug withdrawal from resistant cells

NCI-H358 cells were seeded into T75 flasks (1 × 10^6^ cells per flask) and the following day the media was replaced with media supplemented with the indicated concentration of inhibitor. Resistant cell populations were generated using a constant IC_90_ high concentration of inhibitors (2.63 µM ARS1323, 830 nM ARS1620, 87 nM selumetinib, 2.34 µM AZD4785). Media changes were carried out every 2–3 days for small molecule inhibitors and every 4–5 days for AZD4785 until the cells reached 80% confluency. The cells were then trypsinised and re-seeded at the original density and aliquots of the remaining cells were prepared for liquid nitrogen storage. The development of emerging resistance was monitored by shifts in the cell viability dose responses using IncuCyte® Nuclight Rapid Red Reagent (Sartorius). To examine the effects of drug withdrawal from resistant cells, the day after seeding cells were washed with media only and then treated with media containing 0.1% DMSO. Cells were split as required or media changed every 2–3 days until confluent.

### Incucyte analysis

For monitoring inhibitor combination assays, NCI-H358 resistant cells were seeded in 384-well Corning TC-treated plates at a density of 2000 cells per well in 40 µl of 10% FBS RPMI-1640 medium. Plates were incubated overnight in the IncuCyte S3 at 37°C inside a humidified incubator at 95% O_2_ and 5% CO_2_. The following day cells were treated with corresponding IC_90_ inhibitor concentrations, A 5-fold dilution series of combination inhibitors were applied, giving a final assay volume of 80 µl. Cells were incubated in the IncuCyte for 4 days following treatment and images were collected every 6 h. Cells were analysed using the IncuCyte S3 2020B software. For triple inhibitor combination assays in naïve cells, NCI-H358 cells were seeded in 96-well Corning TC-treated plates at a density of 1000 cells per well in 100 µl of 10% FBS RPMI-1640 medium. Plates were incubated overnight in the IncuCyte S3 at 37°C inside a humidified incubator at 95% O_2_ and 5% CO_2_. The following day NCI-H358 cells were dosed manually with inhibitor concentrations to give a final assay volume of 150 µl. Cells were incubated in the IncuCyte S3 for 14 days following treatment and were re-dosed with fresh media and inhibitors every 5 days. Images were collected every 6 h and cells were analysed using the IncuCyte S3 2020B software.

### Cell viability assays

Cells were seeded in 96-well black walled TC-treated plates for 2D culture and 96-well black walled ultra-low attachment plates for 3D culture. Cells were seeded at a density of 1000 cells per well in 60 µl of 10% FBS RPMI-1640 medium, allowed to adhere or form spheroids overnight and the following day a 3-fold dilution series of indicated inhibitors were applied to give a final volume of 80 µl per well. Cell viability was measured using the CellTiter-Glo. 2.0 assay (Promega) according to manufacturer's instructions. An amount of 100 µl of CellTiter-Glo. reagent was added to each well. For 2D cell cultures the plate was mixed on an orbital shaker at 300 rpm for 2 min and incubated at room temperature for 10 min, whereas for 3D culture mixing was extended to 5 min followed by a 25 min incubation. The luminescence was recorded using the GloMax Plate Reader.

### Preparation of cell lysates for SDS–PAGE and western blotting

Cell culture medium was removed, and cells were washed twice with ice-cold PBS and the lysed for 10 min with ice-cold RIPA buffer (10 mM Tris pH7.5, 150 mM NaCl, 1% sodium deoxycholate, 0.1% SDS, 1% Triton X-100) supplemented with protease inhibitor cocktail (1 : 250 ratio) (Sigma–Aldrich) and PhosSTOP phosphatase inhibitors (1 : 100 ratio) (Roche). Lysates were collected using a cell scraper and clarified by centrifugation (13 000×***g*** at 4°C for 20 min). The supernatant was transferred to a fresh tube and the protein concentration determined using the Pierce™ BCA assay according to manufacturer's instructions. Samples were prepared for Western blotting in 5× sample buffer (15% SDS, 321.5 mM Tris–HCl pH 6.8, 50% glycerol, 16% 2-Mercaptoethanol, 1.25% Bromophenol blue) and diluted in RIPA lysis buffer to the desired concentration. Samples were heated at 95°C for 5 min and stored at −20°C.

### SDS–PAGE and western blotting

Lysates were separated by SDS–PAGE (XCell Sure Lock System, ThermoFisher). Samples were run on pre-cast NuPAGE® 4–12% Bis-Tris gels before transfer onto 0.45 µM pore size nitrocellulose membrane. The membrane was blocked in 5% (w/v) powered milk in Tris-buffered saline with Tween (TBS-T: 10 mM Tris pH 7.4, 150 mM NaCl, 0.1% (v/v) Tween 20) for 1 h and then probed with primary antibodies against AKT, phospho-AKT S473, ERK1/2, phospho-ERK1/2 T202/Y204, phospho-MEK1/2 S217/S221, EGFR, phospho-EGFR Y1068, ERBB2, phospho-ERBB2 Y1221/Y1222, FGFR1, phospho-FGFR1 Y653/Y654, PDGFRβ, phospho-PDGFRβ Y771 (Cell Signaling), DUSP6 (Abcam) and β-actin (Proteintech) diluted in 5% milk TBS-T overnight at 4°C with agitation. Membranes were then washed in TBST for 3 × 5 min and then probed with IRDye® secondary antibodies (LI-COR) in 5% milk TBS-T for 1 h at RT. Membranes were again washed in TBST for 2 × 5 min followed by a final TBS (10 mM Tris pH 7.4, 150 mM NaCl) wash. Detection was performed on the LI-COR Odyssey® CLx imaging system.

### Ras activity assay

Ras activity was measured using Ras Activation Assay BioChem Kit (Cytoskeleton Inc) according to the manufacturer's instructions. Briefly, 200 µg of RIPA buffer cell lysate was added to 30 µl of beads and incubated with rotation at 4°C for 1 h. Following washing, samples were analysed by SDS–PAGE and Western Blot using the total sample eluate.

### Quantitative reverse transcription PCR (qRT-PCR)

Cell lysates were used directly in a one-step qRT-PCR reaction using the iTaq Universal SYBR® Green One-Step Kit (Bio-Rad) according to the manufacturer's instructions and a CFX real-time PCR detection system (Bio-Rad). The following primer sequences were used: *KRAS* forward: 5′-GATGTACCTATGGTCCTAGTAG-3′, KRAS reverse: CATCATCAACACCCTGTCTTG) *DUSP6* forward: CGGAAATGGCGATCAGCAAG, *DUSP6* reverse: TGTGCGACGACTCGTATAGC, and *ACTIN* forward: CACCTTCTACAATGAGCTGCGTGTG, *ACTIN* reverse: ATAGCACAGCCTGGATAGCAACGTAC. Reactions were carried out in triplicates, gene expression was normalized to *ACTIN* housekeeping gene Ct values and quantified using the comparative Ct (−ΔΔCt) method.

### Preparation of RNA samples for RNA-sequencing

Cells were trypsinized and resuspended in PBS. 2 × 10^5^ cells were pelleted and resuspended in 1 ml TRIzol reagent. Cell lysates in TRIzol were stored at −80°C until required. Cells were thawed at room temperature and 200 µl of chloroform added, and samples centrifuged (12 000×***g*** for 15 min). The upper aqueous phase was removed and transferred to a fresh Eppendorf tube. An equal volume of 70% ethanol was added, and samples were immediately loaded onto a RNeasy Mini Spin Column (Qiagen) and RNA extraction performed according to manufacturer instructions. RNA was eluted in 30 µl of RNase free water. A Nanodrop 1000 spectrophotometer was used to assess RNA purity and concentration. RNA integrity was measured using the RNA 6000 Nano Kit (Agilent) according to the manufacturer's instructions and samples run on the Agilent 2100 Bioanalyzer.

### RNA-Sequencing quantification library

RNA-sequencing and bioinformatics was performed by BGI Tech Solutions (Hong Kong) using the Illumina-HiSeq2500/4000 platform. Sequencing reads which contained low-quality, adaptor polluted and high unknown base (N) reads were first filtered out. Clean reads were mapped to the reference genome using HISAT2 [52]. After genome mapping StringTie was used to reconstruct transcripts and with genome annotation novel transcripts were identified using Cuffcompare [53]. Novel transcripts were then merged with reference transcripts to achieve a complete reference. Clean reads were mapped using Bowtie2 [54] and gene expression levels calculated with RSEM [55]. Based on gene expression levels, Poisson distribution algorithms were used to detect differentially expressed genes (DEGs). GO analysis was performed using DAVID [56]. RNAseq raw and processed data available via NCBI Gene Expression Omnibus (GEO) database, GSE206867.

## Data Availability

The authors declare that all data that support the findings of this study are available within the paper and supplementary files. RNAseq raw and processed data available via NCBI Gene Expression Omnibus (GEO) database, GSE206867

## Open Access

Open access for this article was enabled by the participation of University of Liverpool in an all-inclusive *Read & Publish* agreement with Portland Press and the Biochemical Society under a transformative agreement with JISC.

## Abbreviations

DEG: differentially expressed gene
FBS: fetal bovine serum
GEO: Gene Expression Omnibus
PBS: phosphate-buffered saline
PI3K: PtdIns 3-kinase

## References

[1] Prior, I.A., Hood, F.E. and Hartley, J.L. (2020) The frequency of Ras mutations in cancer. Cancer Res. 80, 2969–2974 10.1158/0008-5472.CAN-19-3682 32209560 PMC7367715

[2] Simanshu, D.K., Nissley, D.V. and McCormick, F. (2017) RAS proteins and their regulators in human disease. Cell 170, 17–33 10.1016/j.cell.2017.06.009 28666118 PMC5555610

[3] McGranahan, N., Favero, F., de Bruin, E.C., Birkbak, N.J., Szallasi, Z. and Swanton, C. (2015) Clonal status of actionable driver events and the timing of mutational processes in cancer evolution. Sci. Transl. Med. 7, 283ra254 10.1126/scitranslmed.aaa1408 PMC4636056 25877892

[4] Hruban, R.H., Goggins, M., Parsons, J. and Kern, S.E. (2000) Progression model for pancreatic cancer. Clin Cancer Res. 6, 2969–2972 PMID: 10955772

[5] Prior, I.A., Lewis, P.D. and Mattos, C. (2012) A comprehensive survey of Ras mutations in cancer. Cancer Res. 72, 2457–2467 10.1158/0008-5472.CAN-11-2612 22589270 PMC3354961

[6] Haigis, K.M., Cichowski, K. and Elledge, S.J. (2019) Tissue-specificity in cancer: the rule, not the exception. Science 363, 1150–1151 10.1126/science.aaw3472 30872507

[7] Seo, K.Y., Jelinsky, S.A. and Loechler, E.L. (2000) Factors that influence the mutagenic patterns of DNA adducts from chemical carcinogens. Mutat. Res. 463, 215–246 10.1016/S1383-5742(00)00047-8 11018743

[8] Wiesweg, M., Kasper, S., Worm, K., Herold, T., Reis, H., Sara, L. et al. (2019) Impact of RAS mutation subtype on clinical outcome-a cross-entity comparison of patients with advanced non-small cell lung cancer and colorectal cancer. Oncogene 38, 2953–2966 10.1038/s41388-018-0634-0 30568222

[9] Canon, J., Rex, K., Saiki, A.Y., Mohr, C., Cooke, K., Bagal, D. et al. (2019) The clinical KRAS(G12C) inhibitor AMG 510 drives anti-tumour immunity. Nature 575, 217–223 10.1038/s41586-019-1694-1 31666701

[10] Hallin, J., Engstrom, L.D., Hargis, L., Calinisan, A., Aranda, R., Briere, D.M. et al. (2020) The KRAS(G12C) inhibitor MRTX849 provides insight toward therapeutic susceptibility of KRAS-mutant cancers in mouse models and patients. Cancer Discov. 10, 54–71 10.1158/2159-8290.CD-19-1167 31658955 PMC6954325

[11] Moore, A.R., Rosenberg, S.C., McCormick, F. and Malek, S. (2020) RAS-targeted therapies: is the undruggable drugged?. Nat. Rev. Drug Discov. 19, 533–552 10.1038/s41573-020-0068-6 32528145 PMC7809886

[12] Awad, M.M., Liu, S., Rybkin, I.I., Arbour, K.C., Dilly, J., Zhu, V.W. et al. (2021) Acquired resistance to KRAS(G12C) inhibition in cancer. N. Engl. J. Med. 384, 2382–2393 10.1056/NEJMoa2105281 34161704 PMC8864540

[13] Hong, D.S., Fakih, M.G., Strickler, J.H., Desai, J., Durm, G.A., Shapiro, G.I. et al. (2020) KRAS(g12c) inhibition with sotorasib in advanced solid tumors. N. Engl. J. Med. 383, 1207–1217 10.1056/NEJMoa1917239 32955176 PMC7571518

[14] Skoulidis, F., Li, B.T., Dy, G.K., Price, T.J., Falchook, G.S., Wolf, J. et al. (2021) Sotorasib for lung cancers with KRAS p.G12C mutation. N. Engl. J. Med. 384, 2371–2381 10.1056/NEJMoa2103695 34096690 PMC9116274

[15] Misale, S., Fatherree, J.P., Cortez, E., Li, C., Bilton, S., Timonina, D. et al. (2019) KRAS g12c NSCLC models Are sensitive to direct targeting of KRAS in combination with PI3K inhibition. Clin. Cancer Res. 25, 796–807 10.1158/1078-0432.CCR-18-0368 30327306

[16] Lou, K., Steri, V., Ge, A.Y., Hwang, Y.C., Yogodzinski, C.H., Shkedi, A.R. et al. (2019) KRAS(g12c) inhibition produces a driver-limited state revealing collateral dependencies. Sci. Signal. 12, eaaw9450 10.1126/scisignal.aaw9450 31138768 PMC6871662

[17] Ryan, M.B., de la Cruz, F., Phat, F., Myers, S., Wong, D.T., Shahzade, E. et al. (2020) Vertical pathway inhibition overcomes adaptive feedback resistance to KRAS(G12C) inhibition. Clin. Cancer Res. 26, 1633–1643 10.1158/1078-0432.CCR-19-3523 31776128 PMC7124991

[18] Xue, J.Y., Zhao, Y., Aronowitz, J., Mai, T.T., Vides, A., Qeriqi, B. et al. (2020) Rapid non-uniform adaptation to conformation-specific KRAS(G12C) inhibition. Nature 577, 421–425 10.1038/s41586-019-1884-x 31915379 PMC7308074

[19] Fedele, C., Li, S., Teng, K.W., Foster, C.J.R., Peng, D., Ran, H. et al. (2021) SHP2 inhibition diminishes KRASG12C cycling and promotes tumor microenvironment remodeling. J. Exp. Med. 218, e20201414 10.1084/jem.20201414 33045063 PMC7549316

[20] Solanki, H.S., Welsh, E.A., Fang, B., Izumi, V., Darville, L., Stone, B. et al. (2021) Cell type-specific adaptive signaling responses to KRAS(G12C) inhibition. Clin. Cancer Res. 27, 2533–2548 10.1158/1078-0432.CCR-20-3872 33619172 PMC9940280

[21] Santana-Codina, N., Chandhoke, A.S., Yu, Q., Malachowska, B., Kuljanin, M., Gikandi, A. et al. (2020) Defining and targeting adaptations to oncogenic KRAS(G12C) inhibition using quantitative temporal proteomics. Cell Rep. 30, 4584–4599.e4584 10.1016/j.celrep.2020.03.021 32234489

[22] Tanaka, N., Lin, J.J., Li, C., Ryan, M.B., Zhang, J., Kiedrowski, L.A. et al. (2021) Clinical acquired resistance to KRAS(G12C) inhibition through a novel KRAS switch-II pocket mutation and polyclonal alterations converging on RAS-MAPK reactivation. Cancer Discov. 11, 1913–1922 10.1158/2159-8290.CD-21-0365 33824136 PMC8338755

[23] Akhave, N.S., Biter, A.B. and Hong, D.S. (2021) Mechanisms of resistance to KRAS(G12C)-targeted therapy. Cancer Discov. 11, 1345–1352 10.1158/2159-8290.CD-20-1616 33820777 PMC8178176

[24] Janes, M.R., Zhang, J.C., Li, L.S., Hansen, R., Peters, U., Guo, X. et al. (2018) Targeting KRAS mutant cancers with a covalent G12C-Specific inhibitor. Cell 172, 578–589.e17 10.1016/j.cell.2018.01.006 29373830

[25] Ross, S.J., Revenko, A.S., Hanson, L.L., Ellston, R., Staniszewska, A., Whalley, N. et al. (2017) Targeting KRAS-dependent tumors with AZD4785, a high-affinity therapeutic antisense oligonucleotide inhibitor of KRAS. Sci. Transl. Med. 9, eaal5253 10.1126/scitranslmed.aal5253 28615361

[26] Caunt, C.J., Sale, M.J., Smith, P.D. and Cook, S.J. (2015) MEK1 and MEK2 inhibitors and cancer therapy: the long and winding road. Nat. Rev. Cancer 15, 577–592 10.1038/nrc4000 26399658

[27] Davies, B.R., Logie, A., McKay, J.S., Martin, P., Steele, S., Jenkins, R. et al. (2007) AZD6244 (ARRY-142886), a potent inhibitor of mitogen-activated protein kinase/extracellular signal-regulated kinase kinase 1/2 kinases: mechanism of action in vivo, pharmacokinetic/pharmacodynamic relationship, and potential for combination in preclinical models. Mol. Cancer Ther. 6, 2209–2219 10.1158/1535-7163.MCT-07-0231 17699718

[28] Boned Del Rio, I., Young, L.C., Sari, S., Jones, G.G., Ringham-Terry, B., Hartig, N. et al. (2019) SHOC2 complex-driven RAF dimerization selectively contributes to ERK pathway dynamics. Proc. Natl Acad. Sci. U.S.A. 116, 13330–13339 10.1073/pnas.1902658116 31213532 PMC6613145

[29] Jones, G.G., Del Rio, I.B., Sari, S., Sekerim, A., Young, L.C., Hartig, N. et al. (2019) SHOC2 phosphatase-dependent RAF dimerization mediates resistance to MEK inhibition in RAS-mutant cancers. Nat. Commun. 10, 2532 10.1038/s41467-019-10367-x 31182717 PMC6557854

[30] Caunt, C.J. and Keyse, S.M. (2013) Dual-specificity MAP kinase phosphatases (MKPs): shaping the outcome of MAP kinase signalling. FEBS J. 280, 489–504 10.1111/j.1742-4658.2012.08716.x 22812510 PMC3594966

[31] Fernandes Neto, J.M., Nadal, E., Bosdriesz, E., Ooft, S.N., Farre, L., McLean, C. et al. (2020) Multiple low dose therapy as an effective strategy to treat EGFR inhibitor-resistant NSCLC tumours. Nat. Commun. 11, 3157 10.1038/s41467-020-16952-9 32572029 PMC7308397

[32] Duncan, J.S., Whittle, M.C., Nakamura, K., Abell, A.N., Midland, A.A., Zawistowski, J.S. et al. (2012) Dynamic reprogramming of the kinome in response to targeted MEK inhibition in triple-negative breast cancer. Cell 149, 307–321 10.1016/j.cell.2012.02.053 22500798 PMC3328787

[33] Lee, S., Rauch, J. and Kolch, W. (2020) Targeting MAPK signaling in cancer: mechanisms of drug resistance and sensitivity. Int. J. Mol. Sci. 21, 1102 10.3390/ijms21031102 PMC7037308 32046099

[34] Nussinov, R., Tsai, C.J. and Jang, H. (2021) Anticancer drug resistance: an update and perspective. Drug Resist. Updat. 59, 100796 10.1016/j.drup.2021.100796 34953682 PMC8810687

[35] Zhao, Y., Murciano-Goroff, Y.R., Xue, J.Y., Ang, A., Lucas, J., Mai, T.T. et al. (2021) Diverse alterations associated with resistance to KRAS(G12C) inhibition. Nature 599, 679–683 10.1038/s41586-021-04065-2 34759319 PMC8887821

[36] Fedele, C., Ran, H., Diskin, B., Wei, W., Jen, J., Geer, M.J. et al. (2018) SHP2 inhibition prevents adaptive resistance to MEK inhibitors in multiple cancer models. Cancer Discov. 8, 1237–1249 10.1158/2159-8290.CD-18-0444 30045908 PMC6170706

[37] Anderson, G.R., Winter, P.S., Lin, K.H., Nussbaum, D.P., Cakir, M., Stein, E.M. et al. (2017) A landscape of therapeutic cooperativity in KRAS mutant cancers reveals principles for controlling tumor evolution. Cell Rep. 20, 999–1015 10.1016/j.celrep.2017.07.006 28746882 PMC5567854

[38] Gelatti, A.C.Z., Drilon, A. and Santini, F.C. (2019) Optimizing the sequencing of tyrosine kinase inhibitors (TKIs) in epidermal growth factor receptor (EGFR) mutation-positive non-small cell lung cancer (NSCLC). Lung Cancer 137, 113–122 10.1016/j.lungcan.2019.09.017 31568888 PMC7478849

[39] Omerovic, J., Hammond, D.E., Clague, M.J. and Prior, I.A. (2008) Ras isoform abundance and signalling in human cancer cell lines. Oncogene 27, 2754–2762 10.1038/sj.onc.1210925 17998936 PMC2557550

[40] Kessler, L., Malik, S., Leoni, M. and Burrows, F. (2021) Potential of farnesyl transferase inhibitors in combination regimens in squamous cell carcinomas. Cancers (Basel) 13, 5310 10.3390/cancers13215310 34771475 PMC8582567

[41] Untch, B.R., Dos Anjos, V., Garcia-Rendueles, M.E.R., Knauf, J.A., Krishnamoorthy, G.P., Saqcena, M. et al. (2018) Tipifarnib inhibits HRAS-driven dedifferentiated thyroid cancers. Cancer Res. 78, 4642–4657 10.1158/0008-5472.CAN-17-1925 29760048 PMC6095730

[42] Nichols, R.J., Haderk, F., Stahlhut, C., Schulze, C.J., Hemmati, G., Wildes, D. et al. (2018) RAS nucleotide cycling underlies the SHP2 phosphatase dependence of mutant BRAF-, NF1- and RAS-driven cancers. Nat. Cell Biol. 20, 1064–1073 10.1038/s41556-018-0169-1 30104724 PMC6115280

[43] Molina-Arcas, M., Moore, C., Rana, S., van Maldegem, F., Mugarza, E., Romero-Clavijo, P. et al. (2019) Development of combination therapies to maximize the impact of KRAS-G12C inhibitors in lung cancer. Sci. Transl. Med. 11, eaaw7999 10.1126/scitranslmed.aaw7999 31534020 PMC6764843

[44] Hillig, R.C., Sautier, B., Schroeder, J., Moosmayer, D., Hilpmann, A., Stegmann, C.M. et al. (2019) Discovery of potent SOS1 inhibitors that block RAS activation via disruption of the RAS-SOS1 interaction. Proc. Natl Acad. Sci. U.S.A. 116, 2551–2560 10.1073/pnas.1812963116 30683722 PMC6377443

[45] Ostrem, J.M., Peters, U., Sos, M.L., Wells, J.A. and Shokat, K.M. (2013) K-Ras(G12C) inhibitors allosterically control GTP affinity and effector interactions. Nature 503, 548–551 10.1038/nature12796 24256730 PMC4274051

[46] Bozic, I., Reiter, J.G., Allen, B., Antal, T., Chatterjee, K., Shah, P. et al. (2013) Evolutionary dynamics of cancer in response to targeted combination therapy. eLife 2, e00747 10.7554/eLife.00747 23805382 PMC3691570

[47] Tolcher, A.W., Khan, K., Ong, M., Banerji, U., Papadimitrakopoulou, V., Gandara, D.R. et al. (2015) Antitumor activity in RAS-driven tumors by blocking AKT and MEK. Clin. Cancer Res. 21, 739–748 10.1158/1078-0432.CCR-14-1901 25516890 PMC4335074

[48] Adjei, A.A., Cohen, R.B., Franklin, W., Morris, C., Wilson, D., Molina, J.R. et al. (2008) Phase I pharmacokinetic and pharmacodynamic study of the oral, small-molecule mitogen-activated protein kinase kinase 1/2 inhibitor AZD6244 (ARRY-142886) in patients with advanced cancers. J. Clin. Oncol. 26, 2139–2146 10.1200/JCO.2007.14.4956 18390968 PMC2718422

[49] Das Thakur, M. and Stuart, D.D. (2013) The evolution of melanoma resistance reveals therapeutic opportunities. Cancer Res. 73, 6106–6110 10.1158/0008-5472.CAN-13-1633 24097822

[50] Barbolosi, D., Ciccolini, J., Lacarelle, B., Barlesi, F. and Andre, N. (2016) Computational oncology–mathematical modelling of drug regimens for precision medicine. Nat. Rev. Clin. Oncol. 13, 242–254 10.1038/nrclinonc.2015.204 26598946

[51] Bairoch, A. (2018) The cellosaurus, a cell-line knowledge resource. J. Biomol. Tech. 29, 25–38 10.7171/jbt.18-2902-002 29805321 PMC5945021

[52] Kim, D., Langmead, B. and Salzberg, S.L. (2015) HISAT: a fast spliced aligner with low memory requirements. Nat. Methods 12, 357–360 10.1038/nmeth.3317 25751142 PMC4655817

[53] Pertea, M., Pertea, G.M., Antonescu, C.M., Chang, T.-C., Mendell, J.T. and Salzberg, S.L. (2015) Stringtie enables improved reconstruction of a transcriptome from RNA-seq reads. Nat. Biotechnol. 33, 290–295 10.1038/nbt.3122 25690850 PMC4643835

[54] Langmead, B. and Salzberg, S.L. (2012) Fast gapped-read alignment with bowtie 2. Nat. Methods 9, 357–359 10.1038/nmeth.1923 22388286 PMC3322381

[55] Li, B. and Dewey, C.N. (2011) RSEM: accurate transcript quantification from RNA-Seq data with or without a reference genome. BMC Bioinformatics 12, 323 10.1186/1471-2105-12-323 21816040 PMC3163565

[56] Huang da, W., Sherman, B.T. and Lempicki, R.A. (2009) Systematic and integrative analysis of large gene lists using DAVID bioinformatics resources. Nat. Protoc. 4, 44–57 10.1038/nprot.2008.211 19131956

