# Supplementary material for: Combinatorial approaches for mitigating resistance to KRAS-targeted therapies
Source: Biochem J. 2022 Sep 23;479(18):1985–97. doi: 10.1042/BCJ20220440 (PMC9555794; doi:10.1042/BCJ20220440)
Supplement: Supplementary Material [file BCJ-479-1985-s1.pdf]

Combinatorial approaches for mitigating resistance to KRAS-targeted therapies

Hannah R. Warren[1], Sarah J. Ross[2], Paul D. Smith[2], Judy M. Coulson[1,3] and Ian A. Prior[1,3]

Supplementary Material

FIGURE LEGENDS

Supplementary Figure 1. *Sensitivity of NCI-H358 cells to Ras targeted therapeutics.* Antisense KRAS AZD4785 results in loss of KRAS and a concomitant decrease in Ras-MAP kinase output indicated by decreased DUSP6 expression. Higher sensitivity to drug is observed in 3D spheroid cell culture vs 2D culture following 7 days of incubation. Similar results are observed for the other drugs. Data are means ±SD from three independent experiments. Calculated IC_{50} and IC_{90} concentrations are summarized in the table.

Supplementary Figure 2: *Re-activation of Ras effectors is $KRAS^{G12C}$ specific.* Acute treatments with IC_{90} concentrations of inhibitor were carried out in (A) naïve NCI-H1792 *$KRAS^{G12C}$* cells and (B) naïve NCI-H1793 *$KRAS^{WT}$* cells. Treatments were re-dosed every 48 hours. NCI-H1792 cells exhibited rapid re-activation of Ras effectors following acute inhibitor treatments, whereas NCI-H1793 cells showed minimal changes in pERK levels. Blots are representative of two independent experiments.

Supplementary Figure 3. *Dose responses of combination therapeutics in resistant NCI-H358 cells.* Long-term resistant cell lines were treated with their previous IC_{90} inhibitor doses in combination with dose responses of additional targeted inhibitors.

The change in confluency was recorded following 4 days of inhibitor treatment. Confluence data are means ±SD from at least two independent experiments.

Supplementary Figure 4. *Synergy vs dose response matrices of triple combination therapeutics in resistant cells.* Synergy scores from double inhibitor combination assays were calculated using the Loewe model and plotted against % viability. Areas coloured blue indicate drug combinations that are synergistic. The final doses selected for triple combination analysis in Supplementary Figure 3 are indicated.

Pathway inhibition

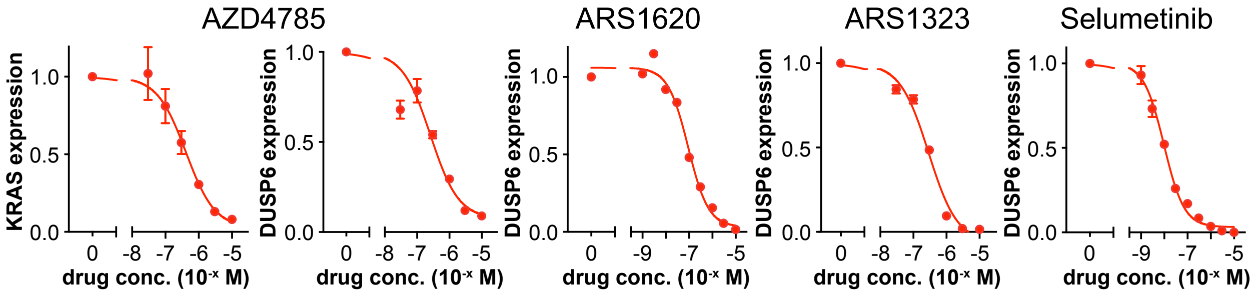

Viability

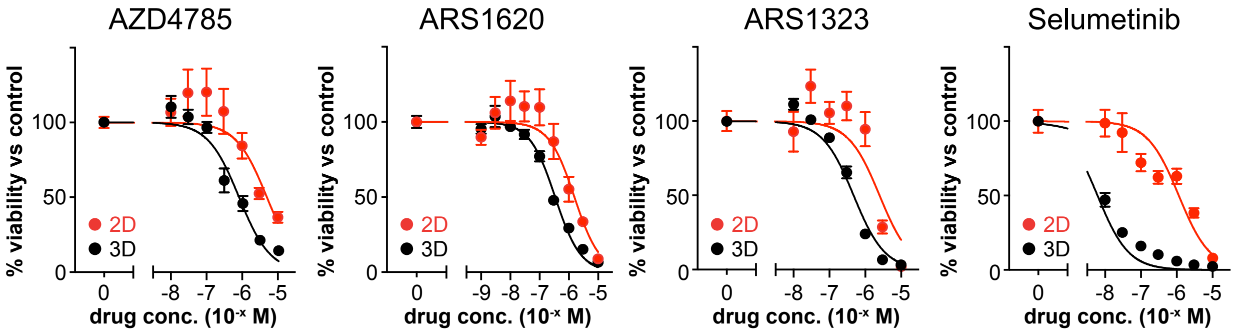

		**Pathway inhibition**		**2D viability**		**3D viability**	
**Inhibitor**	**Target**	**IC50 (μM)**	**IC90 (μM)**	**IC50 (μM)**	**IC90 (μM)**	**IC50 (μM)**	**IC90 (μM)**
AZD4785	KRAS	0.42	3.72	4.95	44.7	0.79	7.08
ARS1620	$KRAS^{G12C}$	0.092	0.83	1.55	13.8	0.34	3.09
ARS1323	$KRAS^{G12C}$	0.29	2.63	2.5	22.4	0.48	4.27
Selumetinib	MEK1/MEK2	0.0097	0.087	1.1	10.2	0.0062	0.055

Supplementary Figure 1

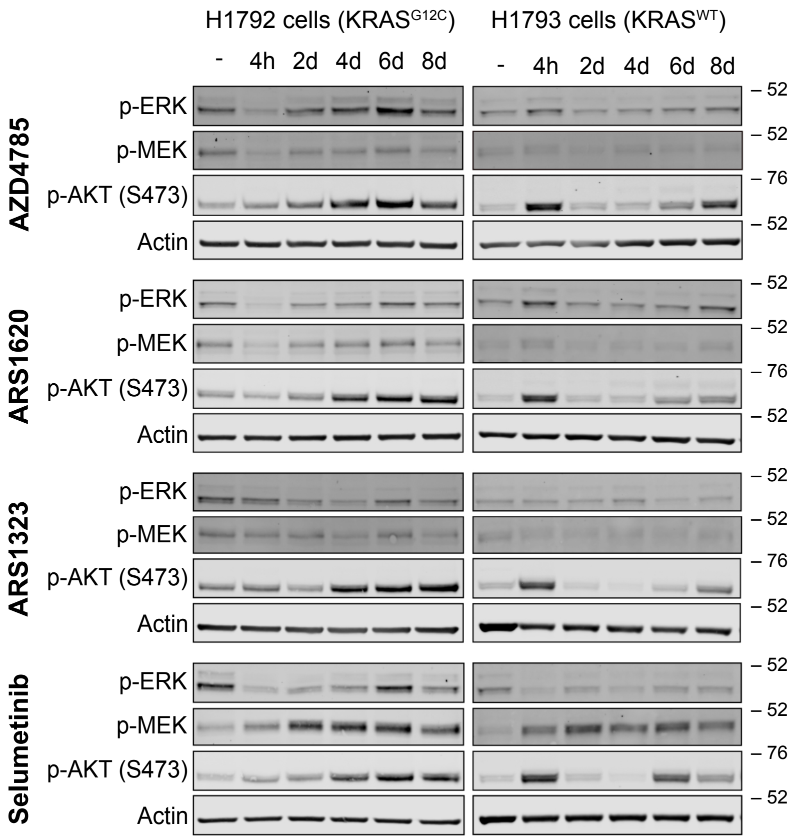

Supplementary Figure 2

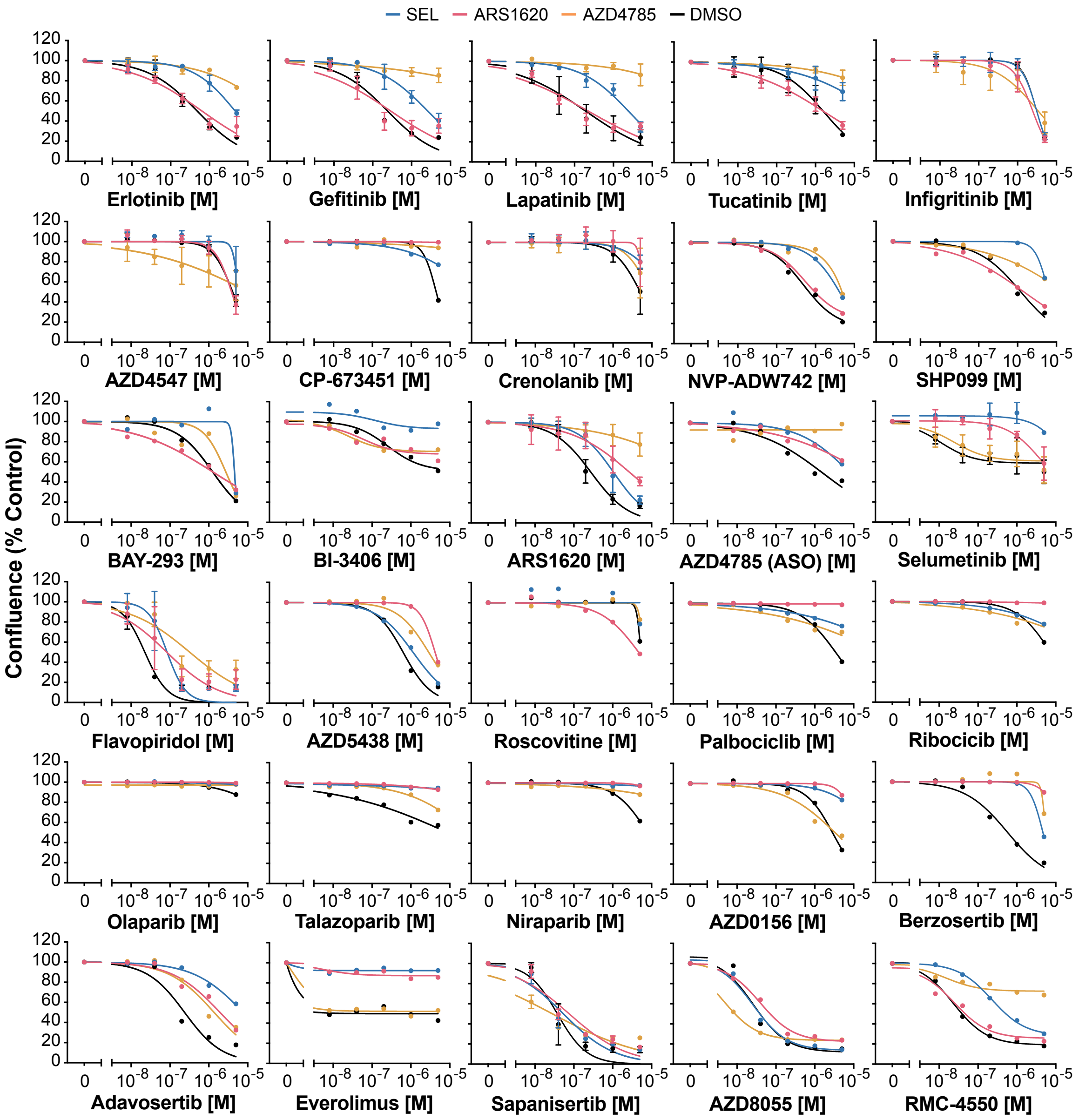

Supplementary Figure 3

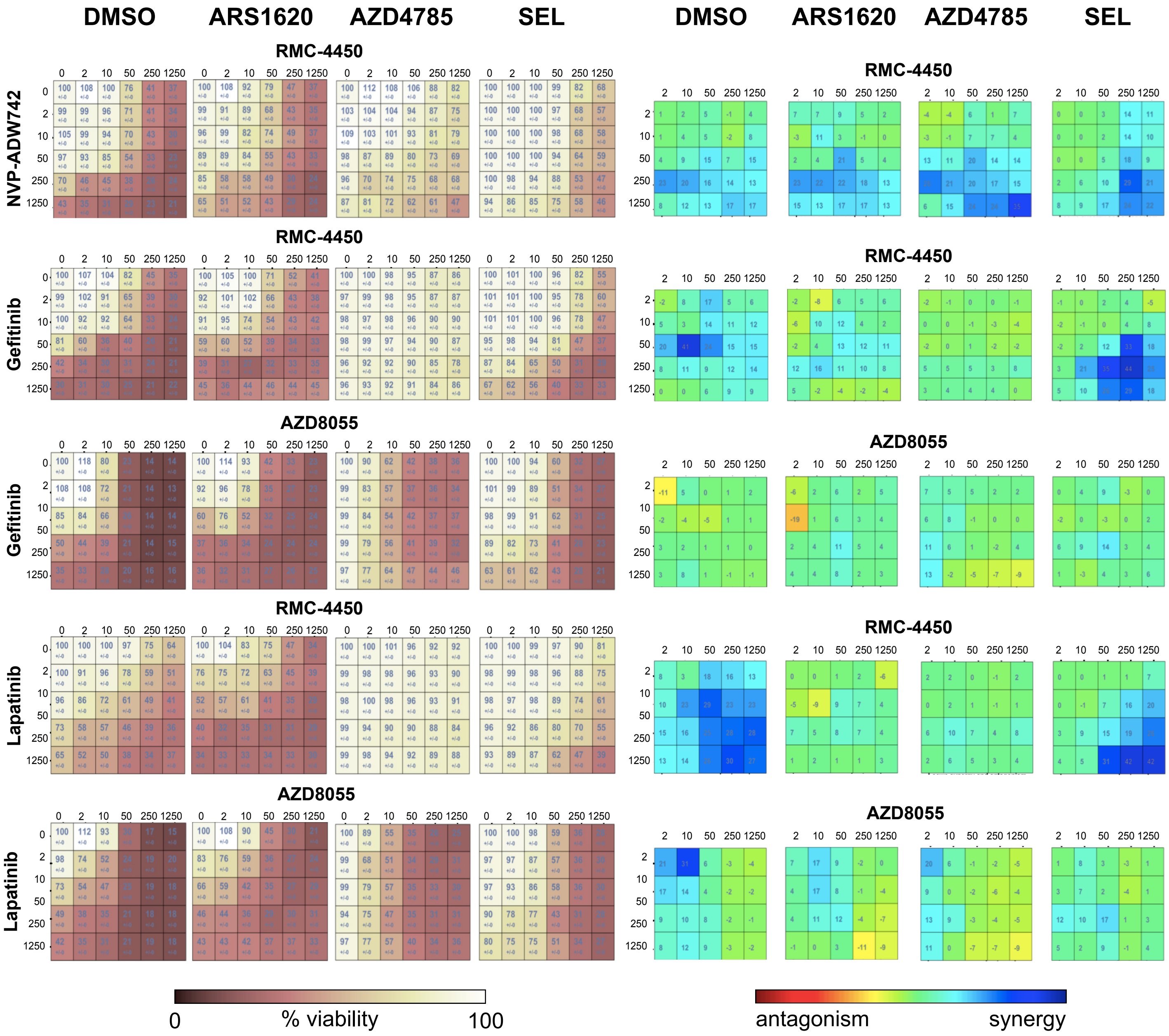

	DMSO	830nM ARS1620	2.34μM AZD4785	87nM SEL
NVP-ADW742	250	50	50	250
RMC-4550	50	50	50	250
Gefitinib	50	250	250	250
RMC-4550	10	10	250	250
Gefitinib	50	50	50	50
AZD8055	50	50	50	50
Lapatinib	50	50	50	250
RMC4550	50	50	50	250
Lapatinib	10	10	10	10
AZD8055	10	10	10	10

Supplementary Figure 4
